# Editorial Notes

**Published:** 1930

**Authors:** 


### Editorial Notes

**Bristol Hospitals Council.**

In November, 1929, the Lord Mayor of Bristol invited a group of citizens interested and experienced in the management of the medical institutions of the city to form a council to advise him and his successors how best to discharge the mayoral obligations towards the medical charities of Bristol. These honourable obligations have arisen out of the Hospital Sunday Fund, which every Lord Mayor for some years past has splendidly maintained. Apart from this fund the appeals of separate charities have always sought the backing of the Lord Mayor as a guarantee to the public that the appeal was justified.

On Tuesday, 4th November, 1930, a meeting organized by the Bristol Hospitals Council was held in the Colston Hall, and a programme of the immediate and pressing needs of the medical charities was laid before the public. The Lord Mayor has undertaken, with the support of the Hospitals Council, to launch an appeal for a single scheme to embrace extensions and improvements at the various voluntary hospitals, so as to avoid a multiplicity of appeals which might appear conflicting and prove difficult for the individual citizen to respond to.

A brief epitome of the work desired to be carried out is as follows :—

New Hospital at Winford, providing 100 additional beds.

Entire reconstruction and enlargement of the Eye Hospital, providing over 50 additional beds.

Additions to the Royal Infirmary, providing 30 additional beds.

Additions to the General Hospital, providing 14 additional beds.

Reconstruction and additions to the Children's Hospital, providing new Out-Patient Department and a new Nurses' Home.

Enlargement of Winford Orthopædic Hospital, providing 60 more beds.

Additions to the Homœopathic Hospital and enlarged Out-Patient Department.

It is estimated that a sum of not less than £250,000 will be required to cover the cost of these works.

**Hospital Amalgamation.**

THERE is one point most welcome in this new movement. A close co-operation between the Royal Infirmary and the General Hospital is at last contemplated. The committees of those two institutions have agreed to maintain and staff as a single unit the new hospital at Winford, transferring to Winford such of the patients admitted to the Infirmary and Hospital who are deemed to require open-air treatment. It is further proposed to establish at the Infirmary a radium institute with a staff drawn from the staffs of the Infirmary and Hospital. In this way a return is promised to the admirable and harmonious fusion which succeeded so well during the War, when the two staffs worked together in the Second Southern General Hospital.

It was greatly regretted when at the end of the

War the combined staff was broken up and the two institutions had to pursue their ways separately. A vigorous attempt was made by the Clinical Board of the University to arrange some form of amalgamation between the two institutions. The late Mr. H. H. Wills and other members of the University Council and of the committees of the Infirmary and Hospital supported the project with enthusiasm. Indeed, Mr. H. H. Wills offered a sum of £100,000 to carry it into effect. But the time was not then ripe, and his generous offer was rejected. Now the omens are more favourable and a *rapprochement* is actually within sight. Perhaps the dream of celebrating the centenary of the Bristol Medical School in 1933 by breaking down the barriers between the Infirmary and the Hospital may come to pass after all. When it does, the pioneer efforts of Mr. H. H. Wills must not be forgotten. The most urgent medical need of Bristol to-day is the provision of *one teaching hospital of the first rank* which shall be adequate to the requirements and worthy of the traditions of our city.

**"The Lancet" Commission on Nursing.**

*The Lancet* announces that it has appointed a Commission on Nursing, over which Lord Crawford and Balcarres has consented to preside. The terms of reference of the Commission are to inquire into the reasons for the shortage of candidates, trained and untrained, for nursing the sick in general and special hospitals throughout the country, and to offer suggestions for making the service more attractive to women suitable for this necessary work.

Any organization or individual wishing to provide evidence for the Commission to consider, or to make proposals relating to the improvement of conditions of service of the nursing profession, is asked to communicate with the Hon. Secretary at *The Lancet* Offices, 7 Adam Street, Adelphi, London, W.C.

**Annual Dinner of the Society.** THE Second Annual Dinner of the Bristol Medico-Chirurgical Society was held at the Berkeley Café, on Thursday, 13th November.

The President (Professor D. C. Rayner, F.R.C.S.) occupied the Chair and sixty-two members and guests attended. The evening was most enjoyable, and the Dinner was voted a great success.

---

